# Exploring resident experiences of person-centred care at mealtimes in long-term residential care: a rapid ethnography

**DOI:** 10.1186/s12877-022-03657-5

**Published:** 2022-12-13

**Authors:** Megan Davies, Franziska Zúñiga, Hilde Verbeek, Sandra Staudacher

**Affiliations:** 1 Nursing Science, Department of Public Health, University of Basel, Bernoullistrasse 28, 4056 Basel, Switzerland; 2 Department of Health Services Research, Care and Public Health Research Institute, Maastricht University, 6229 GT Maastricht, The Netherlands; 3 CURAVIVA Schweiz, Zieglerstrasse 53, 3000 Bern 14, Postfach 1003 Bern, Switzerland

**Keywords:** Older adults, Aged Care, Care home, Nursing home, Dining, Qualitative research

## Abstract

**Introduction:**

Poor nutrition is a common ongoing problem in long-term residential care, often resulting in reduced quality of life. Previous research has concluded that the content of the meal, dining environment, service style and general atmosphere all add to the mealtime experience, suggesting that person-centred mealtimes are optimal. However, knowledge about which elements of person-centred care can be achieved in a mealtime setting in a given context is currently lacking. We aimed to understand the mealtime experience in long-term residential care by exploring (missed) opportunities for person-centred care in different settings.

**Methods:**

As part of the TRANS-SENIOR research network, rapid ethnographies, were conducted across multiple sites (including interviews, observations and informal conversations), in a long-term residential care home in the UK, Switzerland and the Netherlands between October 2020 and December 2021.

**Results:**

Following analysis and interpretation of observations, interviews and informal conversations, the following themes were developed where either successfully achieved or missed opportunities for person-centred moments were observed: 1) considering the setting, 2) listening to and implementing resident choice, 3) enabling residents to help/care for themselves and others, 4) providing individualised care in a communal setting, and 5) knowing the person in the past and present. Residents experienced moments of participatory choice, interaction, independence and dignity, but opportunities for these were often missed due to organisational or policy constraints.

**Conclusions:**

There are opportunities for person-centred moments during the mealtime, some of which are taken and some missed. This largely depended on the setting observed, which includes the overall environment (size of dining area, seating arrangements etc.) and allocation of staff resources, and the level of resident involvement in mealtimes, from preparation to the actual activity.

## Background

Mealtimes in long-term residential care (LTRC) offer a sense of normalcy and structure for residents, while providing an opportunity for social interaction with other residents and care staff. According to previous research, staff have recognised that mealtimes are critical to the health and wellbeing of the residents. Along with residents and family members, they feel strongly about the importance of this aspect of life in LTRC [1, 2]. Poor mealtime experiences can lead to nutritional deficiencies, often resulting in malnutrition and reduced quality of life as well as a higher risk of falls and frailty for residents [2, 3]. People living with dementia are at particular risk of poor nutritional intake, which contributes to diminished physical health as well as quality of life. This is, among other things, due to increased behavioural symptoms such as agitation during mealtimes [4]. Additionally, an unsupportive physical environment, which is, e.g., too loud or overstimulating, can be challenging for people living with dementia and reduce their food intake during mealtimes [5]. Person-centred care (PCC), defined as “an approach to practice established through the formation and fostering of therapeutic relationships between all care providers, patients and others significant to them in their lives”, has been suggested as a way of improving mealtimes in LTRC [6]. Person-centred mealtimes can support personhood, as promoted by Kitwood [7], encourages resident autonomy and affirms resident identity through social encounters and comfort [8]. However, certain barriers to this, such as regulations and policy, organizational cultures and routines, staffing issues, and the physical environment can cause staff to feel pressured to complete the ‘task’ of mealtimes quickly and efficiently to the detriment of the mealtime experience [1, 2, 9].

Research suggests that providing more person-centred mealtimes in LTRC allows for a more personalised and homelike experience, improving resident quality of life [1]. The World Health Organization defines quality of life using four domains: (a) physical health; (b) social relationships; (c) psychological health; and (d) environment [10–13]. This means meeting the physical, emotional and social needs of residents [5]. Providing a person-centred mealtime experience considers the context and preferences of an individual, looking at the whole experience to provide a holistic approach to care. Mealtimes can provide a sense of identity for residents, particularly when residents have the opportunity to be involved and are provided choice, as should be the case with a person-centred approach [14]. The success of a person-centred dining experience is primarily measured by how much choice residents have, not only in the food offered, but also where people sit and who with, for example [12]. Although, research has found that residents are given the opportunity to voice their preferences in LTRC, these preferences are not always acted upon [15]. Including resident voices in LTRC research is integral to successfully improving care practices and it is important to ensure this happens [1, 14].

Several difficulties are encountered in providing person-centred care in an institutional setting [2]. For example, residents with visual impairments commented that staff should describe exactly what the food being offered was in order for their needs to be met, but staff often only described the type of food, such as ‘soup’ or ‘meat’ when trying to meet a schedule [14]. Boelsma, Baur [14] also found that LTRC residents felt a sense of community with their fellow residents, which was important to them, but at the same time, they needed to feel like an individual within the environment [14]. Hunter et al. (2016) found that the environment itself is important for resident choice, showing that a ‘person-directed’ environment is positively associated with resident autonomy [16]. Furthermore, Keller, Syed [17] stated that the overall environment is not only relational to staff ability to deliver PCC, but can also reduce resident autonomy if it is too ‘institutional’ [17].

Improving the mealtime experience by adding person-centred elements can improve nutritional intake as well as sustaining the identity and dignity of residents; particularly those living with dementia [1, 12]. Little information is available on cross-national experiences during mealtimes, which might add insight into which elements of PCC can be achieved in a mealtime setting across different European contexts. Additionally, research exploring person-centred mealtime experiences are rarely presented from a resident’s perspective, which is important to understand. This paper therefore aims to explore the mealtime experiences of residents in LTRC, focussing on opportunities for person-centredness throughout the overall mealtime experience.

## Methodology

### Design

This qualitative study was a rapid ethnography conducted across multiple sites in three different countries. Rapid ethnography allowed rich observational, as well as narrative data to be collected in a condensed time frame, and as a team, to gain a deep understanding of the population and context being studied [18]. This methodology provided the necessary flexibility to adapt to the field and to interact with people and events without a rigidly pre-defined structure of the procedure, following mealtime events as they happened. With this immersive rapid ethnographic approach we could familiarise ourselves with the environment to develop an understanding of daily interactions within each LTRC home, which goes beyond discursive formations [19]. A combination of interviews, observations, informal conversations and limited document analysis were conducted in the UK, Switzerland and the Netherlands as part of a wider project “Tri-National Ethnographic Multi-Case Study of Quality of Life in Long-Term Residential Care” (TRIANGLE). Within the TRIANGLE project, our overarching aim was to explore interrelating factors between PCC and resident quality of life following a permanent transition into LTRC from resident, staff and relative perspectives. For the purpose of this paper, we focus on resident and observational data with regard to mealtime experiences. This is a TRANS-SENIOR research network project (EU Horizon 2020), which aims to optimise transitions into LTRC for older adults.

### Setting and sample

Each LTRC home was purposefully selected as homes striving to provide PCC. No further inclusion or exclusion criteria was imposed on the LTRC homes. The recruitment process was supported in each location by a local organisation with links to the LTRC homes, providing confirmation of the LTRC homes’ reputation concerning PCC. This includes Sheffield Hallam University in the UK, CURAVIVA Schweiz in Switzerland and Maastricht University in the Netherlands. All units within the homes were included. Potential participants, which included residents, staff and relatives, were pre-informed of the study verbally and in writing by a local study coordinator in each country. This included information sheets handed to all potential participants, notices displayed in public spaces in the LTRC homes and verbal communication during recruitment. Following agreement to participate, further relevant study information was provided by the research team. No relationship beyond this informative conversation was established with residents prior to the study commencing.

For each site, we selected a purposeful sample to ensure it would be as diverse as possible within the population of each LTRC home. Selection was based on: gender, age, ethnicity, health situation, strength of social networks (residents and relatives); age, gender, ethnicity, educational background, experience in the care for older persons (staff); and from willingness to take part [20, 21]. Potential participants were selected by the local coordinators in coordination with the research team, discussing diversity of characteristics. A total of 3 residents declined to take part in the study, one due to anxiety about taking part in research and two for undisclosed reasons. For full detail of each study setting, see Table 1.

**Table 1:** Details of each study setting

	**UK SITE**	**SWISS SITE**	**NETHERLANDS SITE**
NO. OF BEDS	Approx. 80	Approx. 120	Approx. 50
SITE LAYOUT	• 2 sites (one residential and nursing, one dementia only) • Site 1 (dementia): 2 floors. Both combination of resident bedrooms and communal space (living/dining room) • Site 2 (Residential & Nursing): 3 floors. Upper 2 floors resident bedrooms, ground floor combination of resident bedrooms and 2 communal spaces (living/dining room)	• 3 floors of resident rooms, each with north and south section of bedrooms and a communal dining area • 1 closed dementia unit (north section of 1^st^ floor) • Activity suite (for group activities), outdoor terrace, physical activity/physiotherapy suite (4^th^ floor) • Separate restaurant (Ground floor) – accessible by residents and visitors	• 3 floors, each with resident bedrooms and a communal area (living/dining area and kitchen) • Separate restaurant/café (ground floor) – accessible by residents, visitors and local guests • Separate guest house for small number of residents with advanced dementia
DINING ROOM LAYOUT	• Site 1: 1 dining room per floor for up to 12 residents (tables seat 4–6 residents) • Site 2: large dining room = up to 30 residents (tables seat 6 residents), small dining room = up to 8 residents), tables seat 4 residents. 2 care staff providing service in both areas simultaneously	• Communal restaurant: up to 60 residents and guests when not under infection control procedures (tables seat 2–4 residents). Designated catering staff serving meals • Small dining areas: 1–2 per floor for up to 18 residents per area (tables seat 2–4 residents). Min. 2 care staff per floor	• Communal restaurant: seats up to 50 residents and guests, tables set for numbers as requested. Designated catering staff serving meals • Small dining areas: 1 per floor for up to 16 residents (one table for up to 12 residents and one seating 4). 3–5 care staff per floor
TOTAL RESIDENTS RECRUITED	• 11 residents, including PLWD • Age range 69 – 99; female: n = 7, male: n = 4	• 14 residents, including PLWD • Age range 69 – 99; female: *n* = 8, male: *n* = 6	• 9 residents, including PLWD • Age range 84 – 93; female: *n* = 5, male: *n* = 4
DATA COLLECTION TIMEFRAME	• August – October 2021 • Early and late shifts (breakfast, lunch and dinner) • Weekdays and weekends	• October 2020 – March 2021 • Early and late shifts (breakfast, lunch and dinner) • Weekdays	• October – December 2021 • Early and late shifts (breakfast, lunch and dinner) • Weekdays
COVID-19 RESTRICTIONS IN PLACE FOR STAFF DURING DATA COLLECTION	• Wearing surgical mask, hand hygiene • Each staff tested 1x/shift (LFT) and 1x/week (PCR)	• Wearing surgical mask (changed to FFP2 mask during cases of COVID-19), hand hygiene	• Masks (surgical) worn only during COVID-19 cases, hand hygiene
COVID-19 RESTRICTIONS IN PLACE FOR RESIDENTS DURING DATA COLLECTION	• If single COVID-19 case on site: resident isolated to room, no access to LTRC home • 10 day quarantine period for new admissions • No COVID-19 cases: no restrictions, but visitors not permitted inside LTRC home	• No restrictions in movement on COVID-free units • Units with COVID-19 positive residents: residents restricted to rooms, unit closed to visitors, residents not allowed to communal dining room • 10 day quarantine for new admissions	• No restrictions in movement No quarantine period required for new admissions • Visitors permitted throughout LTRC home
ETHNOGRAPHIC METHODS USED IN DATA COLLECTION	• Interviews • Observations • Informal Conversations	• Interviews • Telephone Interviews (during COVID-19 isolation) • Observations • Informal Conversations	• Observations • Informal Conversations
RESEARCH TEAM INVOLVED IN DATA COLLECTION	• MD	• NP • SSt • MD • SS	• MD • SSt • KR

*Notes: PLWD* people living with dementia, *LFT* Lateral flow testing, *LTRC* long-term residential care, *PCR* polymerase chain reaction test, *MD* Megan Davies (PhD Student), *NP* Nora Peduzzi (Master’s Student), *SSt* Sandra Staudacher (Postdoc), *SS* Séverine Soiron (Master’s Student), *KR* Katherina Rosteius (PhD Student)

### Data collection

#### Observations and informal conversations

In each setting, we immersed ourselves at different times of the day to observe day-to-day life and daily routines. This included observation of mealtimes, relative visits, activities and physiotherapy as well as shadowing staff members on varying shifts. During this time, natural interactions with residents occurred, providing us with further insight. Informal conversations, which occurred naturally during observational periods, allowed us to gain a deeper level of information with participants in a relaxed environment and an informal manner [22]. Detailed fieldnotes were taken by research team members on site during the observation period and informal conversations and were written up immediately afterwards to ensure a broad view of what was seen and details would not be diluted or misremembered and to allow analysis to begin simultaneously to data collection [23].

#### Interviews

In view of the overall TRIANGLE aim, residents were asked questions based on a broad interview guide, which followed quality of life domains outlined by WHO, during which residents were asked open questions about their experiences of environmental, social relationships and psychological and physical health aspects. We also asked how residents experienced these aspects during COVID-19. This meant that the interview could remain largely unstructured and be adapted to the needs of the person being interviewed. This was particularly important for people living with dementia, to capture their experiences in a less restrictive way. Follow-up interviews were arranged as required to gain a deeper level of understanding, but with most participants only one interview was conducted. Interviews lasted from 20 – 90 min, but on average lasted approx. 1 h. Interview data was audio recorded and transcribed verbatim afterwards to ensure all detail was accurately captured [24]. Interviews were arranged with each participating resident with only the resident and interviewer(s) present and were mostly conducted in the resident’s room or in a private area of the home.

Data saturation can be challenging in ethnographic research due to the broad data collected during the study period and therefore was not an aim of this study [25] Data saturation is increasingly criticised as vague concept in qualitative research, therefore we aimed at rich, contextualised data of each case [26].

### Data analysis and synthesis

Data analysis accompanied data collection from the start and followed an inductive approach. To begin with, an open coding approach was used. Data was coded by one member of the research team using thematic analysis, who highlighted these in the interview transcripts, observation notes and notes taken during analysis discussions [27]. Codes were discussed within the research team, which led to the generation of initial themes. This secondary stage of analysis allowed exploration of significant patterns across the discussed data using MAXQDA [28]. The themes were then discussed and reviewed against the data by the full research team, which led to the definition and naming of the final themes.

### Research team background and skills

The research team involved in data collection included backgrounds in nursing science (SS), social anthropology (SSt, NP), health care policy (KR), and sports science (MD), the latter with previous experience working with the older adults in LTRC. All members of the research team were trained to understand the aim of the study and how to conduct ethnographic research.

### Findings and reflections

During the initial analysis to explore PCC in each of the three locations, it became clear that mealtimes were an integral part of LTRC life and bore importance for residents as well as the overall LTRC home routines. We therefore chose to focus on this element of LTRC living to further understand PCC in each of the three LTRC homes. During discussions about observed person-centred moments experienced at mealtimes, we found times when person-centred moments were successfully achieved, and moments where opportunities for person-centredness were missed. We subsequently developed the following themes: 1) considering the setting 2) listening to and implementing resident choice 3) enabling residents to help/care for themselves and others 4) providing individualised care in a communal setting 5) knowing the person in the past and present. We explore these themes below, using examples from observations, interviews and informal conversations in each site.

### Considering the setting

Each LTRC home provided PCC training and strived to put this into practice. However, to understand the possibilities for PCC at mealtimes in each case, it is important to consider the particular setting. In all three LTRC homes, meals were offered in a large communal dining area, a small communal dining area, or in bedrooms, but with differences observed in each site. See Table 1 for details of the physical dining arrangements in each LTRC home.

#### UK site

In the UK site, mealtimes were at set times each day, to fit with the kitchen assistants who prepared the food, and morning or afternoon activities, including daily activities (washing, dressing etc.) and leisure activities. The same care staff were responsible for serving the meals and assisting residents with mobilising to the dining area as needed. This meant that meals were not served until all residents were seated at the tables. During the mealtime service, the main duty of care staff was to serve food and assist with eating as required; therefore, care activities for residents requiring assistance were halted until food service was complete. For residents eating in their rooms, either by choice or because the resident was bedbound or unable to mobilise to the dining room, one care team member was responsible for collecting and serving food from the kitchen downstairs and assisting with eating when needed. In each case, meals were served pre-plated to order. During this time, nursing staff would provide additional support where possible, however mealtimes coincided with medication rounds, so this had to be completed first for all residents requiring medication.

#### Swiss site

In the Swiss site, unlike the UK site, the larger communal dining area was set up like a restaurant, with designated catering (cooking and serving) staff. The smaller dining areas were positioned on residential floors, which was typically where residents who required assistance with eating would dine. Here we observed at least two care staff members assisting residents during any mealtime, which included helping with eating or making and serving drinks. An additional care staff member on each floor was responsible for assisting bedbound residents eating. As in the UK site, meals were served pre-plated to order. We observed some staff in this Swiss LTRC home eating their own meals at the same time as residents and in an area visible to residents dining in the larger communal restaurant. During this time, other than greeting residents as they passed on their way to the dining area or back to their bedrooms, there was no interaction; specifically, none revolving around care. Data collection was conducted during the peak of the second wave of COVID-19, therefore some practices were not as standard; for example, during this time, staff assisting residents within the smaller communal areas were also required to serve the meals, which they usually would not.

#### Dutch site

The LTRC home in the Netherlands was similar to that in Switzerland in that the larger communal dining area was set up like a restaurant. Although, unless residents were dining with non-residential guests, meals were generally served by floor in the smaller communal dining areas. In this case, care staff were responsible for preparing and serving food to residents as well as assisting with eating for residents who needed help. During mealtimes, 3–5 care staff members were observed to assist with mealtime tasks. Once residents were served their meals, which were served family style for residents to help themselves where able, any care staff not helping residents eat, sat to eat their own meals at the same time, in the same room as the residents. Care staff took it in turns to respond to care needs during this time.

The differences in settings in the three LTRC homes outlined in Table 1 and above were clear when observing each location. The setting itself seemed to influence the potential for person-centredness during mealtimes. The biggest difference was seen in the UK, where low staff numbers and adherence to health and safety requirements acted as barriers to resident choice.

### Listening to and implementing resident choice

Throughout the mealtime experience in each site, choices were made available for residents. We observed choices in menu options, where residents ate and who with, for example. It was clear that offering resident choices was an important element of providing care in each of the LTRC homes. However, resident involvement in choices was not always possible, and certain restrictions, such as dietary requirements, reduced the successful implementation of choice.

#### Menu options

In the UK, a choice of two main course and two dessert options were always provided to residents with a ‘standard’ diet during each meal. However, at times, one option was favoured by residents, causing it to run out. This left both residents and staff unhappy and removed the possibility to choose between dishes. There was no caveat for residents who did not want either option. I was told by a female resident “…they might offer you two things…if you don’t like that or you don’t like that there’s nothing else” A male resident said similarly: “they always come up with the same two items to choose from…”. Residents in the UK site on special diets (either by choice, e.g., vegan, or for medical need, e.g., soft diet) had only one option, as these meals were prepared in smaller quantities. This also meant there was no possibility for additional servings. Similarly, in the Swiss site, two menu options were provided. Although, unlike the UK site, the two menu options always included a meat and vegetarian option. In the Dutch site, the amount of menu choice depended on the meal. For example, during breakfast and lunch, residents were provided with a selection of food to help themselves to, for example breads, cheeses and meats. However, the evening meal was decided and prepared by care staff, depending on available ingredients and the skillset of the ‘chef’, offering only one option. In the Swiss and Dutch sites, residents were offered a wider choice of drinks, with a range of soft drinks and alcoholic drinks available in the Swiss site. The UK site offered a choice of two different flavours of fruit squash, served by staff. Although, wine was planned as a menu option following a discussion during a resident meeting.

#### Portion sizes

In the UK and Swiss sites, meals were pre-plated, leaving no choice in portion size. Meanwhile, the Dutch site served meals ‘family style’ so residents could serve themselves, which provided choice in portion size and extra servings as they wished.

Portion sizes in the UK were often discussed by residents during food service, with regular commentary that the portion sizes were too large. During food service, one male resident stated “each portion they serve here seems huge…almost horse sized!”. Similar comments were heard in the Swiss site, where a female resident explained that portion sizes were too big for her, but seemed too small for a male resident sharing her table: “…he sometimes got the dinner that is not the dinner for a man…you know, so little…and from this I eat only half right?…I eat very little…”. Although on the surface this appeared to be a negative comment, she further explained that the discrepancy in portion sizes led to a meaningful interaction between the two, telling us “…just sometimes, if I’ve had eggs, then I’ve shared them [with him] before…”. A further barrier to portion control in the UK site was when residents on special diets requested additional servings. For example, during an evening meal, a female resident eating a ‘standard’ diet was offered more food. A female resident eating a soft diet on the same table asked if she could also have a second portion, but she was told they had no soft diet food left. She told me “I want more, but I don’t get it”. Even though the individual ‘special’ diet had been provided for the resident, there was no flexibility in portion size, which limited how much involvement in choice this resident could have during her meal.

#### Who to sit with/where to sit

Providing residents with a choice of who to sit with during mealtimes can encourage the development of relationships and social interaction in general between residents. Considering individual seating preferences for residents provides an opportunity for person-centredness during mealtimes. We observed opportunities for this that were taken and missed in each site.

Increased resident dependency seemed to impact choice during mealtimes. For example, in the UK site, residents needing a wheelchair to mobilise were positioned at tables without discussion of where they might like to sit or who with. Meanwhile, independently mobile residents chose to sit where they would like. On one occasion, a newly admitted was wheeled through and the care assistant was heard saying “where will he get the most chat?”. This resident had moved from the dementia to the nursing and residential site in the UK LTRC home as it had been decided that he needed more social interaction with his level of cognition. During an interview with this resident, he spoke about his first experience of communal dining in the nursing and residential site, explaining that he tried to interact with ladies on the table the care assistant had wheeled him to. “…I says ‘good day to you all’…I says ‘my name is’…and I come from…’…nobody spoke!”. Although the care assistant had considered which residents in the room would be most sociable, she did not factor in this gentleman’s hearing impairment. He explained “…nobody talked to me except one old lady, but I’m more deaf now…I think she got tired of herself repeating…so I thought well, I’ll be better off stopping at home…”. Aside from residents with prior relationships spanning before their move to LTRC, mealtime seating tended to be chosen by space availability.

Reduced choice from higher dependency was also observed in the Swiss site, where residents requiring more assistance had less choice of mealtime location than more independent residents. Here, residents requiring full assistance with eating were not given the option of dining in the larger communal restaurant. Instead they ate in either the smaller communal dining areas or in their bedrooms (depending on how limited their overall mobility was). Limitations were also observed within choices provided. For example, in the larger communal dining area, seating was allocated by staff. Although staff based decisions about who would sit where on resident backgrounds to try to sit like-minded residents together. Each resident had a designated seat in the large dining area, so they sat at the same table, with the same people during each meal. One female resident spoke favourably of seating arrangements, telling us “…I had a catholic priest, that was ‘tiptop’…we were able to talk to him about God and the world…next to me I had an older lady…she had climbed 6000 m in Tibet”. Although another resident told us about his more challenging experience with a language barrier between him and his table mate, explaining “…yes, such a pity, she doesn’t know German very well…she can speak Italian well and I can’t speak Italian and she can’t speak German”. This limited the potential for interaction during mealtimes, but could not be changed without staff intervention since seating is allocated without resident input.

Possibilities for choice occurred throughout the mealtime experience. There were moments where choice was offered, such as menu options, but was not extensive, because such options were decided without pre-discussion with residents. Such limitations, which could impact resident autonomy seemed easily fixable within the constraints of the mealtime settings.

### Enabling residents to help/care for themselves and others

There are several opportunities for person-centredness during mealtimes to encourage resident independence following a move into LTRC. For example, enabling residents to serve themselves or providing information to empower residents to make better nutritional, but enjoyable choices. We saw instances of this being facilitated in some areas, but also barriers to this in others.

The Swiss and Dutch sites provided constant resident access to kitchens in the smaller communal dining areas on each floor. We observed residents helping themselves to drinks as they wished in the Dutch site. In the Swiss site, a male resident, who preferred to remain independent in the LTRC home, which he viewed more as a source of support, spoke about his morning routine, which consisted of getting up earlier than other residents. He told us that he ate after ‘doing sport, washing and shaving’, which meant he was “…in the kitchen by half past 5 at the latest…” to get himself “…coffee…and a yoghurt…” unassisted and in his own time. Access to food and drink differed in the UK site. Here residents could only access food and drink provided by the LTRC home when it was prepared/served by staff. Jugs of drinks were available for and accessible by residents in the communal sitting area in the UK site, but no cups were laid out and residents were seemingly unaware of them, so could/did not help themselves. Access to the kitchen, including fridges, was available to staff only.

Some differences were also observed during mealtimes. For example, during one mealtime in the UK site, residents were able to choose from a selection of pre-made sandwiches served from a large tray. However, staff served sandwiches that residents selected, even though it would have been possible for them to take their own. In contrast, in the Dutch site, bread and different fillings were laid out on the table for residents to choose from and make their own sandwiches. This created opportunities for interaction between residents and in fact led to residents offering each other food and at times assisting each other, for example passing bread to one another. Additional interactions were also observed in the Dutch site during one breakfast, when a younger male resident was observed providing tea and coffee to all residents and taking empty pots to be refilled. This seemed to give the resident a sense of purpose during the meal. Likewise, in the UK, a (semi-independent) female resident requested, and was given fruit juice instead of squash, and was often able to serve herself from the carton. Anytime this resident was able to pour her own drink, she offered and served it to other residents sitting near her, creating further independence and interactions between residents.

Providing opportunities for residents to help themselves and other residents encouraged further interaction between residents. Furthermore, these interactions provided an opportunity for staff to learn more about individual residents. For example, residents who demonstrated a background in care related work. Additionally, residents were able to maintain a higher level of independence by helping themselves. Without this, expectations on staff appeared to increase during mealtimes, causing additional staff burden on staff and resident discontent.

### Providing individualised care in a communal setting

PCC considers the individual needs of each resident in order to improve quality of life, however, we observed that providing care in a communal setting challenged this concept. For example, in some cases, mealtimes were provided at set times each day to accommodate organisational routines or activities, hindering the possibility for PCC.

Organisational resources, such as staffing, were observed to challenge the possibilities for PCC; more so in some cases than others. This was mostly observed within the UK site, particularly during mealtimes where care staff provided a dual role, as mentioned above. Although, there were designated kitchen staff, who cooked and plated the food, staff worked in a ‘domestic’ rather than a ‘care’ capacity. This created tension between staff and residents. For example, one noticeably stressed care assistant had to tell a resident asking to be taken to the bathroom “…you’ll have to wait ‘til after dinner, I’m on my own…”. At times this also led to distress amongst residents, with others sitting at the same table trying to get staff attention saying “…come and get her, she really needs the toilet…” and “…you need to take her to the toilet now – help her!”.

In the UK site, two care staff members typically served food to around 20 residents sitting between the larger and smaller dining areas, including assisting with eating as needed. A nurse or the activities coordinator would help when possible, particularly when understaffing meant only there was only one care assistant to serve and assist with meals. However, nurses had to complete medication rounds, which coincided with mealtimes first. In addition to food service, care staff were responsible for mobilising dependent residents to the dining area. This often caused a delay between residents being taken to eat and food being served, which caused dissatisfaction among residents. During one lunch, two female residents discussed the anticipated wait, with the first asking “I wonder what time we’ll eat today…” and the other replying “…well…anytime between now and half one…” at that point it was 12:15 pm. On residential floors, one care staff member was responsible for serving and assisting with meals for residents eating in their bedrooms. This occasionally caused residents to wait for their meal to arrive or for assistance with eating, if required. One female resident, who preferred to eat in her bedroom rather than a group environment discussed her experiences, explaining “…I seem to have a very late breakfast this morning, I think they forgot about me…”.

Residents in the UK site were either encouraged to move or assisted to move to the dining area. It was immediately observed that residents with lower mobility had less autonomy than fully independent residents. Residents requiring lower assistance to mobilise (frame or stick but with supervision due to falls risk) were often observed being offered a wheelchair, on the surface providing further choice and assistance. However, one female resident, who needed assistance to mobilise with a stick explained that she preferred to walk to maintain mobility, saying “…if you don’t move them, you lose them…” but that staff “…don’t have time because I take so long…”. Therefore, she often opted for the wheelchair offered, even when she preferred to walk.

One resident in the Swiss site preferred to eat in the larger communal restaurant, despite being fully dependent on assistance to mobilise. Once there, she was provided meals that meant she could eat without (or with minimal) assistance, for example pre-cut food and a straw with her drinks. However, she was dependent on staff to move from the residential floor to the dining area. We observed this resident waiting for some time to move to the restaurant downstairs while staff remaining on the residential floor were occupied serving and assisting with meals.

Providing personalised care is difficult during a communal activity, which is part of a structured routine. We observed longer wait times and less choice available for more dependent residents during communal activities. During mealtimes, it appeared difficult for staff to cater to each resident individually. However, there are possibilities for person-centred moments within this; for example, ensuring residents can still dine in all communal areas even when requiring assistance or allowing the time to encourage residents to walk when they wish to and are able.

### Knowing the person in the past and present

To successfully deliver PCC, it is important to know the whole person, which includes understanding who they were prior to moving in to LTRC. In each site, efforts were made to learn resident histories, which included asking residents and family members about food and drink preferences. Staff in each site explained that this was considered during menu planning. However, in most cases, residents weren’t actively involved in menu planning.

In the UK and Swiss sites, the overall menu planning is done by catering staff without pre-discussing options with residents. Catering staff have little interaction with residents, apart from brief interactions during mealtimes. This limited knowledge of resident likes and dislikes. One male resident discussed food he ate prior to moving into the LTRC home, explaining “…I don’t have this type of food…” describing the food here as “…bloody awful…”. A male resident in the Swiss site spoke similarly about how meals were not as he would have eaten them at home, saying “…that’s all I don’t like to eat. I’ve never had it at home either”. Menu planning was slightly different in the Dutch site, where residents have input on grocery lists when food supplies are needed. However, as mentioned above, the evening meal is still decided by staff.

In each site, staff encouraged residents to eat in a communal setting rather than their bedrooms and to interact with other residents, particularly if staff knew a resident like to be sociable. However, this wasn’t always easy for residents. A male resident in the UK site, who struggled to eat without spilling due to some physical disability said during an interview “…I like being with people, but they don’t necessarily like being with me…”. He seemed sad explaining this. This resident relied fully on staff to move around the home as he is in a wheelchair and had residual disabilities following a stroke. Staff were not seen to ask him whether he would prefer to eat somewhere else, but in his interview he made it clear that he likes to be around people. A female resident, who opted to eat in her bedroom though being largely independent explained that she felt self-conscious eating in front of others now as she has false teeth. Staff were not observed to encourage her to dine in communal areas.

Knowing the person goes beyond just knowing what food or drink each resident prefers. In each site, we observed evidence of staff knowing a resident, for example how they took their tea or coffee, or who they preferred to sit with as was seen in the UK site. In the Dutch site, staff had knowledge of which foods residents like to buy from the store. In the Swiss site, staff had learned resident personalities, for example residents who were chattier during mealtimes. As with other elements of PCC, more dependence often resulted in less option for the resident, which sometimes meant things weren’t exactly as the resident would like them to be. Altered physical or mental states of residents since moving into the LTRC home sometimes meant their preferences had changed. Therefore, it is important that staff get to know the person as they were prior to moving into LTRC, but also the person they have become while living there.

## Discussion

Mealtimes are an integral part of LTRC living, which contribute to the physical health of residents as well as their overall quality of life. During this research, we explored mealtime experiences from resident perspectives, including interactions with and actions of staff, in three different LTRC homes across three different European countries. We found that opportunities for person-centred moments were presented throughout mealtimes, some of which were taken and some missed in view of the following themes: 1) considering the setting, 2) listening to and implementing resident choice, 3) enabling residents to help/care for themselves and others, 4) providing individualised care in a communal setting, and 5) knowing the person in the past and present.

Contextual challenges can, as previous literature suggests, prevent successful implementation of PCC [29, 30]. The three LTRC homes within this study had differing operating styles within different regulatory contexts, which either made opportunities for PCC easier to put into practice or not. Differing restrictions across the three sites, resulting from the ongoing COVID-19 pandemic, created further contextual and procedural disparities during the period of data collection. Opportunities for person-centredness were seen most in the Dutch site, where staff were encouraged to be flexible with the preparation and service of mealtimes to meet resident preferences as much as possible. This approach follows guidance to implementing PCC in previous research, which suggests that flexible working is a pre-requisite to PCC [31]. Furthermore, this is in line with the ‘Dutch national quality framework’ established in 2017 [32]. The fact that the communal dining spaces for residents were smaller in the Dutch site facilitated staff to provide a more person-centred mealtime experience as they had higher staff to resident ratio and staff were able to oversee without being overstretched. This supports research by Keller, Syed [17], who noted that staffing and working environments are related to staff ability to adopt PCC. The UK site demonstrated the most restrictive dining experience, which was largely due to low staffing resources, with care staff also acting as catering staff. This also resulted from the regulatory context, in which staff had to adhere to strict health and safety policy as well as individual resident care plans, leaving little room for spontaneity. Organisational factors linked with staffing and governance have previously been negatively associated with variables such as autonomy and personhood of residents as well as knowing the person [16]. This could explain why less PCC opportunities were seen to be taken in the UK site, even when opportunities were presented. For example, residents could have served themselves with the pre-made sandwiches if staff did not need to note food intake in resident notes. Strict adherence to health and safety over resident preference has previously been noted as a barrier to PCC adoption by McCormack, Dewing [29], who described safety and infection control procedures as ‘adding to depersonalisation’ in care. It should also be noted that restrictions imposed during the COVID-19 pandemic are suggested to have led to more task-focused mealtimes in LTRC, leading to lower satisfaction among residents [33]. However, it is also important to note that responses to PCC practices are both objective and subjective; formed by organisational cultures, but also by how individual staff members perceive person-centredness [34]. Health and safety, which was observed as a barrier to PCC in the UK are also, to some extent, a matter of interpretation. Individual staff behavior can differ, even within the same regulations. Adjustments in individual perceptions, but also organizational culture (underlying values, norms and attitudes) that determine behavior are needed for change to happen.

Our results demonstrated moments where choice was provided and implemented, but also moments where choice was provided, but not implemented, which has previously been noted as a problem in LTRC research [15]. For example, the lack of resident involvement in meal planning in most cases meant that although residents were always offered two options at mealtimes in the UK and Swiss sites, they were not always offered the *right* choices for them. Rather than simply offering a choice, PCC is about ensuring residents are fully involved in the care they receive with shared decision-making; which has been found to allow for high quality resident care [35]. In the Dutch site, residents were able to select and prepare some of their meals, including involvement in planning shopping lists. Other settings in the Netherlands also encourage residents to actively participate in cooking and preparation of evening meals, with staff and residents preparing meals together. Regardless resident ability, they can experience smells and engage in food preparation, which is believed important for PCC and feeling at home [36]. Enabling a greater level of resident engagement in choice has been suggested in previous research as important for providing continuity following a move into LTRC as well as maintaining a sense of identity for residents within a communal setting [14, 37]. Furthermore, encouraging residents to be actively involved in their care and environment provides meaningful engagement for residents [35]. The UK site appeared the most concerned with timings and routines at mealtimes as well as health and safety measures, which immediately restricted how involved residents could be in the mealtime experience. Previous research shows that this rigidity in routines that adhere to more traditional care restricts the possibility to provide anything other than ‘usual care’, making PCC less possible [31]. This can also lead to a more institutional feel in the mealtime setting, which research suggests can reduce resident autonomy [17]. Providing care should not overshadow independent resident choice, even though this must also be balanced with providing both care and choice in a communal environment [37].

Our results highlight the dilemma in providing individualised care within a communal setting, as has also been found in previous research [2, 38]. At mealtimes, we saw a group of relative strangers with different, often complex needs and coming from different living situations come together to dine as a group. As Ettelt, Williams [38] found when speaking to LTRC managers, individualising care was a time-consuming process, which always took longer than catering for the whole group. In a setting with low staff resources, i.e. the UK site, this makes putting PCC into practice incredibly challenging, even if well intended, which risks resident quality of life. It seems that the more flexible approach (i.e., used in the Dutch site), where residents were able to select and serve their own food in a communal setting, allowed for more personalised mealtimes, even in a group setting. However, the importance of tailoring individual needs may cause professional dilemmas for staff [39]. Successful PCC requires staff to meet the individual needs of each resident, which includes care needs [6]. This includes adhering to specialist diets, such as the soft diets seen in the UK site or restricting foods for medical or safety reasons, such as choking. However, this immediately restricts staff from providing freedom of choice to residents. In all three sites, more dependent residents, or those with reduced communication were given less opportunity for choice and involvement; which, although challenging, could be improved with further staff training in ‘reactive care’ [35].

By looking specifically at mealtimes as an activity within LTRC, this study shows that it is difficult to fully implement PCC in a communal setting. It is important to note that in these settings, the communal dining area was one large space for all residents who wished to dine together, where in the Dutch site, communal dining was split by floor. Even though in the Netherlands, residents could dine on different floors depending on who they wanted to eat with, the number of residents dining at the same time was notably smaller than we observed in the UK and Swiss sites. This provided more opportunity to comply with health and safety requirements while also taking more opportunities for PCC. Additionally, there was less possibility for residents to become overwhelmed or overstimulated, which has been suggested as a contributing factor in reducing nutritional intake and quality of life during mealtimes [5]. As has been seen in previous research, this suggests that the local context is integral to successful PCC [16].

Mealtimes are an significant part of LTRC living, which contribute to both the physical health of residents as well as their overall quality of life. By providing person-centred mealtimes, the overall resident experience can be improved. The difficulty is knowing how to make mealtimes, which are an unarguably communal activity within LTRC, person-centred and furthermore, what this means to residents. We saw within our study that opportunities for person-centred moments were presented throughout mealtimes, some of which were taken and some missed. With small adaptions, such as including residents in meal planning or adjusting the environment to accommodate more dependent residents, the mealtime experience could become more person-centred and inclusive of residents, which would contribute to their quality of life.

### Methodological considerations

This study was conducted in a single LTRC home per country and aimed to explore concepts within specific settings. We can only compare across the individual environments seen, so we cannot state whether organisational restrictions or flexibility are a consequence purely of regulations and requirements at government level or due to organisational structures. In addition, changes to data collection procedures due to the COVID-19 pandemic meant that less observation was conducted in the Swiss site during mealtimes and in the Dutch site, only observations and informal conversations were conducted, with no accompanying interviews. This meant that we could only decipher the person-centred moments taken or missed during mealtimes from conversations with residents or subjectively through observations respectively. Restrictions to travel meant that only one (British) team member was able to collect data in the UK site, which limited the multi-cultural insight during observations and interviews possible in the other sites. Although regular team discussions were held during this time with the full research team to minimise the impact of this. Additionally, due to time constraints, we were only able to conduct weekend observations in the UK rapid-ethnographic study. Although, discussion of mealtimes across the three settings included observational data from weekdays for consistency. While this study begins to understand how opportunities for PCC can be taken or missed and where this is important from a resident perspective, it would be important to conduct such a study in multiple LTRC homes in each country to fully understand the different contexts and related barriers to PCC at mealtimes in LTRC. For example, country specific regulations and regulatory bodies and expected measures resulting from this, including staff ratios and levels of training.

## Conclusion

Throughout the mealtime experience, there are opportunities for PCC. Even where opportunities for PCC are taken, they tend to lean towards fulfilling the trained criteria for PCC, rather than providing pure person-centred moments for residents during mealtimes in LTRC. By emphasising the process of care to focus more on the resident than health and safety requirements, which has begun in some cases, a more person-centred mealtime experience could be achieved. Although, a greater understanding of how such individualised care can be provided in a communal setting and within specific local contexts is needed.

## Data Availability

The datasets generated and/or analysed during the current study are not publicly available due to ethical reasons to protect confidentiality of participants and the LTRC homes but are available from the corresponding author on reasonable request.
